# Lessons and tips for designing a machine learning study using EHR data

**DOI:** 10.1017/cts.2020.513

**Published:** 2020-07-24

**Authors:** Jaron Arbet, Cole Brokamp, Jareen Meinzen-Derr, Katy E. Trinkley, Heidi M. Spratt

**Affiliations:** 1 Department of Biostatistics and Informatics, Colorado School of Public Health, University of Colorado-Denver Anschutz Medical Campus, Aurora, CO, USA; 2 Department of Pediatrics, University of Cincinnati College of Medicine, Cincinnati, OH, USA; 3 Division of Biostatistics and Epidemiology, Cincinnati Children’s Hospital Medical Center, Cincinnati, OH, USA; 4 Department of Clinical Pharmacy, Skaggs School of Pharmacy and Pharmaceutical Sciences, University of Colorado, Aurora, CO, USA; 5 Department of Medicine, School of Medicine, University of Colorado, Aurora, CO, USA; 6 Department of Preventive Medicine and Population Health, University of Texas Medical Branch, Galveston, TX, USA

**Keywords:** Machine learning, electronic health record, healthcare research, translational research, research methodology

## Abstract

Machine learning (ML) provides the ability to examine massive datasets and uncover patterns within data without relying on *a priori* assumptions such as specific variable associations, linearity in relationships, or prespecified statistical interactions. However, the application of ML to healthcare data has been met with mixed results, especially when using administrative datasets such as the electronic health record. The black box nature of many ML algorithms contributes to an erroneous assumption that these algorithms can overcome major data issues inherent in large administrative healthcare data. As with other research endeavors, good data and analytic design is crucial to ML-based studies. In this paper, we will provide an overview of common misconceptions for ML, the corresponding truths, and suggestions for incorporating these methods into healthcare research while maintaining a sound study design.

## Introduction

The mandate to adopt electronic health records (EHRs) in 2009 under the Health Information Technology for Economic and Clinical Health Act has resulted in widespread electronic collection of health data [1]. With the increase in the use of electronic medical data comes an increase in the amount of healthcare data that are generated. Such EHR data, insurance databases, and other available clinical data have the potential to aid researchers in solving clinical problems. Machine learning (ML) algorithms are quickly rising to the top of the list of tools that can be used to address such problems. However, how to properly use ML methods in the clinical setting is not widely understood. Liu *et al*. have published an article educating clinicians how to read papers utilizing ML techniques in the medical literature [2]. Nonetheless, understanding the use of ML approaches in healthcare is still needed, especially since precision and accuracy are often key components of solutions to healthcare problems. The intended audience for this manuscript is clinicians and translational researchers interested in learning more about the overall process of ML and predictive analytics, clinicians interested in gaining a better understanding of the working framework for ML so that they can better communicate with the individuals creating such models, and analytics professionals that are interested in expanding their skillset to better understand predictive modeling using healthcare data.

Here, we address some challenges specific to working with EHR data, some best practices for creating a ML model through a description of the ML process, and an overview of various different ML algorithms (with an emphasis on supervised methods but also give mention to unsupervised techniques) that can be useful for creating predictive models with healthcare data. Our goal is not to educate the reader on precisely how to utilize various different ML algorithms but to better understand the process of creating predictive models for healthcare scenarios while using ML techniques. We discuss the pros and cons of several supervised ML methods and also note some additional considerations regarding the use of ML in healthcare that are outside the scope of this manuscript. We finish with a discussion of the limitations of this paper as well as a discussion of the field of ML in healthcare research.

### Challenges Specific to Healthcare Data

Common sources of healthcare data include the EHR and claims submitted to payers for healthcare services or treatments rendered. Healthcare data stem from the need to support patient care, protect clinicians from liability, and to facilitate reimbursement. Healthcare data are not documented or collected for the purpose of research; thus, unique challenges exist when using this type of data for research. One widely used example EHR dataset is Medical Information Mart for Intensive Care III, an openly available database consisting of deidentified health data associated with about 60,000 intensive care unit admission. Different tables within the database compromise information on demographics, vital signs, laboratory test results, medications, and more [3].

It is important to understand the intent and meaning of healthcare data prior to using it as a secondary data source for research. For example, presence of a single diagnosis for coronary artery disease does not necessarily mean a patient was diagnosed with coronary artery disease; rather the diagnosis may be documented because the patient was undergoing evaluation to determine whether they had coronary artery disease and in fact coronary artery disease was ruled out. In this example, an International Disease Classification (ICD) code for coronary artery disease was documented. Such medical nomenclature, including ICD codes (for diagnoses), Current Procedural Terminology (CPT, for procedures), and RxNorm (for medications) are used to document services provided to patients but must be considered within the context of the clinical workflows where they are used. Healthcare data and medical nomenclature must be used in concert with other healthcare data to decipher the context of the clinical situation. In the case of the example above where documentation of an ICD code reflects diagnostic differential and not an actual diagnosis, there are multiple approaches that can be taken to exclude such non-diagnostic cases: including ICD codes documented on more than one encounter separated by a certain time period or including ICD codes in the absence of a CPT code for evaluation within a certain time period. The approach to defining metrics for a given characteristic/variable is complex and varies based on the specific variable. When possible, metrics used to define specific variables should align with approaches published in the literature or formalized computational phenotypes. A computational phenotype is a clinical variable such as a diagnosis that can be determined using structured data and does not require clinician interpretation or manual chart review [4]. Computational phenotypes, such as those available from eMERGE (https://emerge-network.org) or PheKB (www.phekb.org), can be used to overcome the challenges of accurately identifying certain characteristics within EHRs.

Similarly, electronic systems often include more than one field to enter a given data element. Thus, understanding which fields are useful require understanding of the clinical context or workflows. For example, there are often many fields in the EHR where blood pressure can be documented and some may represent patient-reported, which may not be relevant to all research questions. Pertinent fields need to be collapsed and labeled accordingly. In some instances, using a common data model such as the Observational Medical Outcomes Partnership (https://www.ohdsi.org/data-standardization/the-common-data-model/) can help organize healthcare data into a standardized vocabulary. A common data model is especially helpful when integrating healthcare data from disparate sources. One data field in health system A may be used differently than the same field at health system B. Common data models account for such differences, making it easier to accurately pool data from different sources.

While claims data are structured, much of healthcare data within the EHR are unstructured, documented in the form of free-text notes. It is critical to know what data are accurately represented within structured fields and what are not. Data within unstructured documentation often represent a rich source of information and may be necessary to accurately represent the situation. For example, if evaluating disease severity, patient-reported outcomes are often essential, but these are often only documented in unstructured clinical notes. When unstructured data are necessary, text mining and natural language processing (NLP) methods can be used. Such methods to transform unstructured data into structured, analyzable data are becoming more mainstream, but nonetheless do require additional time and can be highly complex.

Healthcare data are also fraught with errors and missingness. Data entry is prone to simple human error including typos and omission in both structured and unstructured data. In some instances, typos and missing data need to be addressed or corrected, but in the context of using healthcare data for ML applications, this is not always ideal. If the intent of a given ML application is to create a predictive model that will continue to use healthcare data, correction of errors would be counterintuitive, given these errors are part of the data and need to be accounted for in the model. The ML models should be created using the actual data they are being created to use, which may include representation of errors and missingness. Further, missingness should not be automatically considered an error. In healthcare, missing data can be an indication of something meaningful and worthy of evaluating. For example, absence of a given provider ordering a urine drug toxicology screen for a patient prescribed high doses of opioids may suggest suboptimal patient care and thus is important to capture. Missing data can also be because the data were irrelevant in a given situation. In healthcare, data are not always missing at random. However, another type of missing data is the product of patients receiving care across different health systems, which is unavoidable, and how it is addressed depends on the research question.

## ML Process

**Fig. 1.**
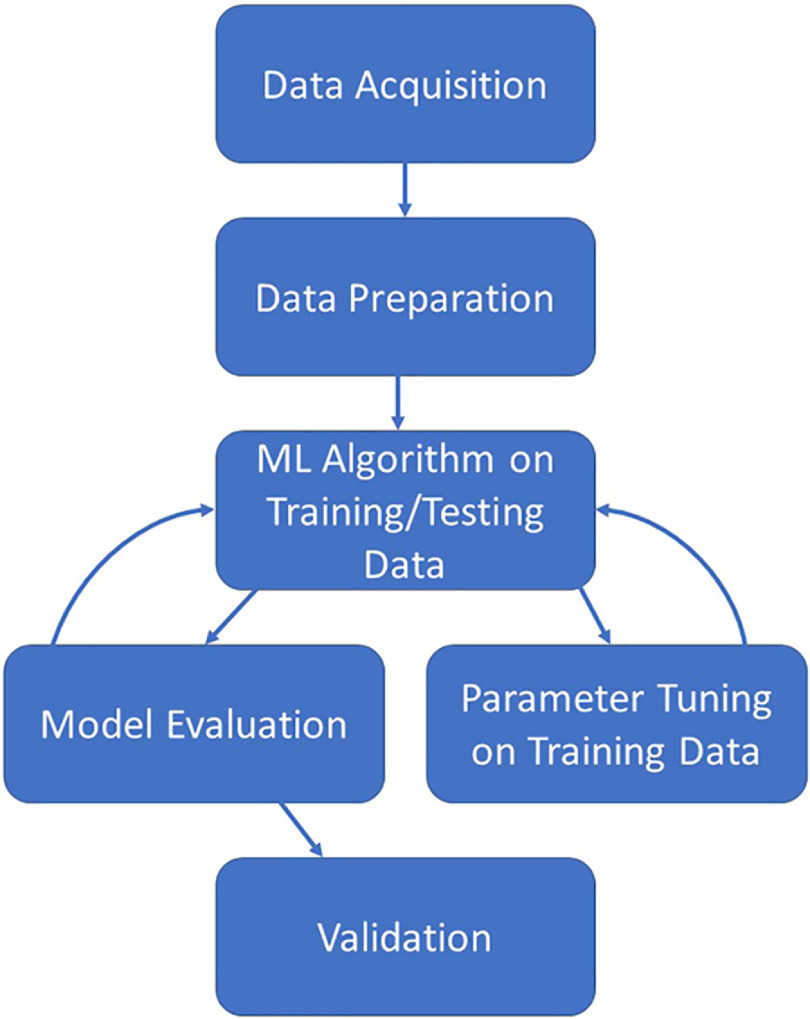
Illustration of the iterative machine learning process.

The creation of a predictive model via ML algorithms is not as straightforward as some in the realm of clinical and translational research would like. However, once the process is fully understood and followed, the creation of a predictive model can be straightforward and rigorous. Fig. 1 illustrates the overall process which is described in this section. The process starts with the acquisition of a worthy dataset (of sufficient size and scope), data preparation which includes steps such as the appropriate treatment of missing data, identifying all data sources/types, and the appropriate treatment of identified errors. Next comes the selection of appropriate ML algorithms for the problem at hand as not all methods work for all types of outcomes. Once a method or methods have been chosen, the model building process can begin. After a suitable model has been built on the training dataset, the model should also be evaluated by examining how well the created model also works on the testing dataset. Finally, the ultimate model should be validated on a separate dataset before it can be used in practice for prediction. Here, we discuss the common steps necessary for creation of a predictive model based on EHR data for clinical use.

### Data Acquisition

The data acquisition step is critically important for the success of the final predictive model. This step often involves writing the appropriate query for an EHR data warehouse to ensure that the correct data for the question at hand are obtained. Before requesting the data, a clear understanding of the research question and appropriate study designs is critical. The research question defines the scope of the data request and the data requested must minimally address the research question. If the question is not clearly defined, it is common to omit key data elements from the request or request more data than necessary. When leveraging EHR data for research, attention must be paid to how the data are generated to minimize important biases that could undermine the study question. Selecting the appropriate study design is foundational and directs the sampling and data analysis necessary to effectively achieve the study aims or objectives. This frequently involves working with an informatician or analyst skilled in creating appropriate queries for relational databases used to store patient information. An important step is also ensuring that the database that one is working with is the most up-to-date version (or if not, knowing that is a limitation of the study).

### Data Preparation

The EHR data acquired can be fraught with errors, whether the data are pulled directly from an EHR software system (such as Epic or Cerner) or compiled by a resident or fellow. Knowing where potential errors may lie is key in knowing how to handle the data downstream. Once potential errors have been identified, they can be properly dealt with and corrected. Data preparation also includes how one handles missing values as creating ML algorithms with vast amounts of missing data can greatly bias the resultant predictive model. Some ML methods implicitly handle missing data, but when they do not, several methods exist for handling missingness. One measure is to remove subjects who have any missing data. This will reduce the sample size of the dataset to ensure only complete information is analyzed, but this approach often introduces selection biases. Another option is to impute the missing data in some fashion. Common imputation methods are replacing the missing value with the mean or median for a given variable, replacing the missing value with zero or the most commonly occurring value for a given variable, or more intensive computational methods such as k-nearest neighbor imputation or methods involving regression. Which method is best to choose depends on the structure of the data and why the value is missing [5]. In addition to cleaning the data, the data preparation step also includes variable selection/feature reduction. While most algorithms will create a model from any number of variables, culling the number of variables chosen for the initial model creation can help ensure the model is not overly complicated or large. Common methods for variable selection include random forests (choose the top x number of variables from the variable importance list), least absolute shrinkage and selection operator [6], principal component analysis [7] (choose the top × number of principal components), stepwise selection procedures, and basic hypothesis testing (choose those variables with a p-value less than a prespecified threshold). Common feature selection methods include filter methods and wrapper methods. Filter methods are where one utilizes some method (a hypothesis test or correlation coefficient for example or linear discriminant analysis) to filter the variables based upon some prespecified value or cutoff [8–10]. Wrapper methods are an iterative process where variables are selected, assessed for model accuracy, and then revised based on performance. Common wrapper methods include forward selection, backward elimination, and recursive feature elimination. The exact number of variables to select is subjective and should be based on the total sample size within the dataset. Additionally, one should also consider model utility in making the decision for how many variables to include in the final model. For instance, if a model is to be clinically relevant and utilized in practice, a physician commonly prefers a model with fewer variables instead of one with 20 as that means less underlying data/testing is needed to inform potential decision-making. These feature reduction and screening methods can be used with or without an outcome in supervised and unsupervised methods, respectively; however, feature screening methods that look at the association between predictors and an outcome must be based on the training data only in order to prevent overfitting/bias [11].

### Choosing a Method

Once your dataset has been checked for errors and missing values have been dealt with, it is time to choose a method for the creation of a predictive model. Not all methods work best for all questions of interest, so it is important to know the format of the outcome variable (is it binary, categorical, continuous?) as well as the format/structure of the independent variables (are they categorical, continuous, normally distributed, correlated, longitudinal?). It is also important to understand if you will be able to use a supervised method or an unsupervised method. Supervised methods assume that the ultimate outcome is known (for instance, this person has Stage 1 cancer vs Stage 2 cancer). Unsupervised methods do not have such assumptions.

### Training/Testing

Despite which method is chosen above, the process of creating a model is similar. Some users are tempted to throw all the data into an algorithm and examine the result. This is less than ideal for creating predictive models that can be useful in the clinic. The reason being is that such a model may be overfit for the data at hand (i.e. the model works really well on the data that were used to create it, but it does not perform well in other scenarios). To avoid overfitting, the model should be created initially on training data and then separately evaluated on testing data. Typically with developing and validating models (training and testing), the dataset is randomly split into a training set (80% of the data) and a testing set (the remaining 20% of the data). While the 80/20 split is commonly used, other splits can be used as well. What is important is that the training set is large enough in sample size to yield meaningful results and also has characteristics that resemble the entire dataset [12]. The model is initially created using only the training set. Evaluation metrics are examined, and then the same model is run using the testing dataset, and evaluation metrics for it are examined. Sometimes, cross-validation techniques are also used on the training dataset.

In general, cross-validation is any approach that leaves some of the data out when creating the initial model and then includes it in a testing fashion [11, 13–17]. This includes splits other than 80/20 (e.g. 60/40), repeated cross-validation (e.g. 80/20 split repeated five times where each set is held out once), bootstrap sampling [18–21], or subsampling. No matter the splitting method, if using a repeated cross-validation technique, the process is repeated multiple times so that different subsets of data are left out for separate runs of the model building process, and the model performance metrics are averaged for each subset of testing data to produce an overall average performance metric [22–26].

The amount of data and overall sample size required for prediction models depend on multiple factors. The sample size considerations are essential in the design phase of prediction modeling techniques as the sample size is dependent on a variety of factors, including the number of outcome events, the prevalence of the outcome or the fraction of positive samples in the total sample size, and the number of candidate predictor variables considered. Additional considerations include the number of classes the model is to predict. If the validation plan for the model is to develop the model on a subset of data and then test the model on the remaining subset, the sample size considerations should account for the split dataset, particularly if the prevalence of the event is low, to ensure the testing set will have ample events to perform.

### Training Set Sample Size Planning for Predictive Modeling

Healthcare databases may contain thousands to hundreds of thousands of available subjects. Given such a large sample size, it may not be necessary to formally estimate the sample size needed for building a prediction model. In other cases, the number of available subjects will be limited and a formal sample size calculation will be more useful. As discussed in Figueroa [27], there are two main approaches to estimate the training dataset sample size needed for building a prediction model: “learning curve” and “model-based” approaches. “Learning curve” methods require preliminary data on a set of subjects with all features/predictors and outcome of interest measured [27–29]. One chooses a specific type of prediction model and estimates the prediction accuracy of the model when varying the sample size by leaving some of the subjects out in an iterative manner. A “learning curve” is then fit to the resulting prediction accuracies compared to the sample size used to fit the model. One can then extrapolate from the learning curve to estimate the sample size needed to obtain a desired level of prediction accuracy. The main downside of this approach is the requirement of substantial preliminary data. In contrast, “model-based” approaches do not require preliminary data but make strong assumptions about the type of data used in the study (e.g. all predictors are normally distributed) [30–32]. Dobbin *et al*. offer an online calculator for estimating the necessary sample size in the training dataset that only requires three user input parameters [31,33]. Despite its strong assumptions, Dobbin *et al*. argue that their method is “conservative” in that it tends to recommend sample sizes that are larger than necessary. McKeigue provide R code for their method which also only requires three user input parameters [32].

### Model Evaluation

Once a model has been created using the training dataset, the model should be evaluated on the test dataset to assess how well it predicts outcomes for new subjects that were not used to build/train the model. The reason for doing this is to ensure that the model is not overfit for the data it was trained on and to also ensure that the model is not biased toward particular values within the training dataset [11]. Random chance alone could have impacted the model fit from the training data, and evaluating the model fit on the testing data can diminish this impact [34,35] Several methods exist for evaluating predictive accuracy, and usually a combination of techniques are used. Evaluation methods for supervised learning with categorical outcomes include assessing discrimination through the area under the receiver operating characteristic curve, which can be obtained by calculating the C-statistic (“Concordance” statistic) and partial C-statistic for the various models [36]. Additional steps include evaluating a confusion matrix which indicates how many individuals the model correctly classifies and how many individuals are incorrectly classified. See Table 1 for a list of prediction accuracy metrics that can be used for particular types of outcomes (e.g. continuous, binary, multi-category, or time-to-event). The chosen evaluation metric would be calculated for the training data and then separately for the testing data. Ideally, the prediction accuracy would be high for both the training and testing datasets. Calibration methods can be used to assess how well the predicted outcomes correlate with the true outcomes, including using scatter plots, reporting a regression slope from a linear predictor, or using the Hosmer–Lemeshow test to compare accurate predictions by decile of predicted probability [37,38]. High accuracy for the training data with low accuracy for the testing data indicate that the model is overfit for the data and will likely not generalize well to other settings. Lastly, special methods should be used when evaluating predictive performance for categorical outcomes with unbalanced classes (i.e. when the number of subjects per class is not approximately equal), such as over-/undersampling, cost-sensitive methods, or precision and recall curves [39,40].

**Table 1.** Metrics for evaluating prediction accuracy for various types of outcomes

Outcome type	Prediction accuracy metric^a^
Continuous	mean squared error (MSE), root mean squared error (RMSE), mean absolute error (MAE), proportion of outcome variance explained (R^2^)
Binary	AUC, C-statistic, sensitivity, specificity, positive predictive value (PPV), negative predictive value (NPV), precision, recall
Categorical with three or more categories	“one-versus-all^b^” versions of metrics for binary outcome, multinomial log-likelihood
Time-to-event	C-statistic, Integrated Brier Score

a Most of the above performance metrics are defined in Kuhn [79]. For machine learning with time-to-event outcomes, Ishwaran [111] used the C-statistic [36], while Bou-Hamad [112] used the Integrated Brier Score [113].

b For a categorical outcome with three or more categories, “one-versus-all” versions of metrics for a binary outcome can be used which assess prediction accuracy for one category versus all other categories combined.

### Parameter Tuning

If the model created does not fit the data well, or if the model is overfit for the training data, parameter tuning is typically the next step in the final predictive model creation process. Parameter tuning involves fine-tuning of parameters that control the overall complexity of the ML model. Hyperparameter tuning is sometimes needed when using various techniques (such as neural networks, support vector machines, or k-nearest neighbors) to optimize the parameters that help define the model architecture. Hyperparameters are parameters that cannot be automatically learned from the model building process, and rather, the user needs to try a grid of values and use cross-validation or some other method to find the optimized value [41]. Olson *et al*. and Lou provide guidance on several different methods for the tuning of hyperparameters and evaluate different methods for different algorithms [42,43]. Tuning of the hyperparameters should only be done utilizing the training data. The model creation process is highly iterative, so once a change is made, the whole process is re-run and re-evaluated.

### Validation

Finally, once a model has been selected as being optimal for both the training and testing datasets, and the variables of interest are selected, the model ultimately needs to be tested a final time on an independent dataset, known as the validation dataset, before it can realistically be used in a clinical setting. This is to ensure the model actually works well in practice, and on a dataset that was independently collected (frequently from a different clinical setting, but with the same initial standards as the training dataset).

## How ML is Used in Clinical and Translational Research

The use of ML with healthcare data can help with several different types of clinical and translational research goals [44–46]. The type of aim or research question will determine which methods are ideal to use and which types of biases and misconceptions will be applicable and should be avoided. The use of ML with healthcare data can be generally classified into four different goals: (1) diagnostics and clinical decision support tools, (2) -ome wide association studies, (3) text mining, and (4) causal inference [47].

*Diagnostics and clinical decision support tools* are designed to give healthcare workers and patients specific information to enhance their clinical care. These tools are often specialized to appropriately filter applicable data based on an individual patient’s condition at a certain time. For example, a clinical decision support tool might utilize the longitudinal health history of a patient with high blood pressure to estimate their risk for a myocardial infarction in the near future. Techniques used for accomplishing this that have been implemented with ML methods include imaging diagnostics, risk stratification, and identification of prognostic subgroups.

*“-ome-wide”* association studies leverage measurements of large numbers of different variants in an -ome system to see if any of them are associated with a disease outcome or health characteristic. These could include the genome, phenome, exposome, microbiome, metabolome, proteome, or transcriptome; often referred to generally as ‘omics data. For example, a genome-wide association study might identify a small list of polymorphisms that are associated with an increased risk for obesity. These types of methods are also used to predict clinical outcomes, like the effectiveness of a drug, or for the identification of gene–gene and gene–environment interactions. See Libbrecht and Noble [48], Ghosh *et al*. [49], and Zhou and Gaillins [50] for a review of ML methods applied in specific types of ‘omics data, or for integrating multiple ‘omics data sources [51,52].

Text mining works by automating data extraction from unstructured clinical notes. The applications can include the identification of patients that might benefit from participation in clinical trials, the removal of protected health information from clinical records to be used for research, the conduct of a systematic literature review, and automated infectious disease surveillance systems [53].

*Causal inference* is the process of making an inference about a specific intervention or exposure with respect to its effect. For example, a causal inference study could be conducted with the goal of determining if taking a specific drug increases the expected lifespan of patients with a certain disease. It is important to consider confounding pathways, or those characteristics that could vary with both the intervention/exposure and the disease outcome, so that we are not misinterpreting an association as a causal effect. Techniques used for accomplishing this that have been implemented with ML methods include propensity score weighting [54], targeted maximum likelihood estimation, marginal structural models, heterogeneous treatment effects, and causal structure learning [55–58].

## Overview of ML Methods

Artificial intelligence (AI) is a scientific field within computer science that focuses on the study of computer systems that perform tasks and solve problems that historically required human intelligence. ML is a subfield of AI that focuses on a special class of algorithms that can learn from data without being explicitly programmed. This is accomplished by making inferences based on patterns found in data. Although some human specification is required, ML algorithms overall require less human input than more traditional statistical modeling (i.e. deciding which variables to include *a priori*). In general, there are three main ways that a machine might learn from data: (1) supervised ML, (2) unsupervised ML, and (3) reinforcement learning.

*Supervised ML*, most often used for prediction modeling, works by mapping inputs to labeled outputs. This is used only when each input used to train the model has a labeled output, for example, an input of longitudinal measurements on a person collected during their hospitalization might be used with a labeled output of in-hospital mortality or unplanned readmission within 30 days of discharge [59]. Supervised ML is also referred to more generally as “predictive modeling,” or “regression” for quantitative outcomes, and “classification” for categorical outcomes. Often, data with labels are used to train a supervised ML algorithm and then the algorithm is used to predict unknown labels for a set of new inputs. Mathematical techniques used to develop supervised ML models include decision trees and random forests [60–62], gradient boosting [63–65], neural networks and deep learning [66–71], support vector machines [72], and regularized/penalized regression [73].

*Unsupervised ML* works by finding patterns in *unlabeled* input data. This category is commonly used for segmentation and clustering problems such as identifying disease “sub-phenotypes” or pattern recognition. For example, a registry of patients diagnosed with asthma might be classified into different subgroups based on their sensitivity to different types of indoor and outdoor allergens, lung function, and presence of wheezing [74]. Mathematical techniques used to develop unsupervised ML models include clustering (hierarchical, k-means), dimensionality reduction, Gaussian mixture models, principal component analysis, and independent component analysis.

*Reinforcement learning* works by improving a sequence of decisions to optimize a long-term outcome through trial and error [75]. It is similar to supervised learning, but a reward mechanism is used in place of a labeled output. This category is often used within healthcare to evaluate and improve policies. For example, a longitudinal series of decisions regarding treatment options must be made by a clinician within the context of managing sepsis (e.g. mechanical ventilation, sedation, vasopressors) [76]. Unlike supervised learning which makes a one-time prediction, the output of a reinforcement learning system can affect both the patient’s future health as well as future treatment options. Often times, a reinforcement learning algorithm will make a short-term decision based on what it believes will result in the best long-term effect, even if that means a suboptimal short-term outcome. Mathematical techniques used to develop reinforcement learning models include neural networks, temporal difference algorithms, Q-learning, and state-action-reward-state-action.

## Pros/Cons of Supervised ML Methods

Fernández-Delgado *et al*. [77] compared the predictive accuracy of 179 ML models (sorted into 17 “families” of different model types) across 121 real datasets and found that random forests were consistently among the best predictive models. Thus, random forests can be recommended as one of the best “off-the-shelf” ML models. Nevertheless, one can never know *a priori* which model will produce the highest predictive accuracy for a given application, thus it is common practice to fit several different ML models and use cross-validation to select a final model that has the highest predictive accuracy. Each ML model has its own pros and cons which should be taken into consideration when deciding which model to use. In addition, when comparing multiple ML models, it is frequently useful to also compare with one or more simple, traditional statistical models such as linear or logistic regression (or penalized versions thereof). For example, a systematic review of 71 clinical predictive modeling papers found no evidence for a difference in predictive accuracy when comparing logistic regression (or penalized logistic regression) with a variety of ML models [78].

Table 2 compares several key properties of different ML models and classical statistical models such as linear or logistic regression. These properties will now be briefly summarized and can be used to help decide which ML model to use for a given problem. For a comprehensive introduction to these ML models, see [79–81].

**Table 2.** General properties of different machine learning models (adapted from Kuhn [12] and Hastie et al. [2]): ✓ = good, **○** = fair, × = poor

Property	Classical models (e.g. linear, logistic regression)	CART (single tree)	Random forests	Boosted trees	Support vector machines	MARS	Neural networks	Penalized regression (LASSO, Ridge, Elastic Net [114])
Allows P > N	×	✓	✓	✓	✓	✓	✓	✓
Automatic nonlinear and interaction effects	×	✓	✓	✓	✓	✓	✓	×
Can handle many irrelevant predictors	×	✓	✓	✓	×	✓	×	✓
#Tuning parameters	0	1	0–1	3	1–3	1–2	≥2	1–2
Robust to outliers/noise in predictors	×	✓	✓	✓	×	×	×	×
Handles missing values in predictors	×	✓^a^	✓^a^	✓^a^	×	**○** ^b^	×	×
Necessary pre-processing^c^	Corr				CS		CS, Corr	CS
Computation time	✓	✓	**○**	×	×	✓	×	✓
Interpretability	✓	✓	×	×	×	✓	×	✓
R software packages	lm(), glm()	partykit, rpart, tree	partykit, randomForestSRC, ranger, randomForest	gbm, xgboost	e1071, kernlab	earth	nnet, tensorflow, keras	glmnet, ncvreg

a Tree-based models can naturally handle missing values in predictors using a method called “surrogate splits” [61]. Although not all software implementations support this, example software that does allow missing predictor values in trees are the rpart [115] and partykit [116] R packages. Other acronyms: “P > N”: the total number of predictors “P” is much larger than the total number of samples “N”; CART: classification and regression tree; MARS: multivariate adaptive regression splines; LASSO: least absolute shrinkage and selection operator.

b In theory, MARS can handle missing values [11]; however, we are not aware of any free software that supports this.

c Corr: remove highly correlated predictors, CS: center and scale predictors to be on the same scale (i.e. cannot naturally handle a mix of categorical and numeric predictors on their original measurement scales).

In contrast to classical statistical models like linear or logistic regression, all of the ML models considered allow the number of predictors (“P”) to be larger than the number of subjects (“N”), that is, “P > N.” In addition, most of the ML models considered automatically allow for complex nonlinear and interaction effects, whereas classical methods (and penalized regression) generally assume linear effects with no interactions. One can manually explore a small number of nonlinear or interaction effects in classical models, but this becomes practically infeasible with more than a handful of predictors, in which case, ML models that automatically allow for complex effects may be more appropriate.

Several ML methods can handle a large number of irrelevant predictors (i.e. predictors that have no relationship with the outcome) without needing to first identify and remove these predictors before fitting the model. Tree-based methods [61] (e.g. classification and regression tree (CART) [82], random forests, boosted trees) use a sequential process that searches through all predictors to find the “optimal” predictor (that best improves the predictive accuracy) to add to the tree at each step in the tree-building process. Multivariate adaptive regression splines (MARS) [83] uses a similar “step-wise” or sequential model building approach. In doing so, these methods automatically search for the most important predictors to include at each step of the model-fitting process, thereby disregarding unimportant predictors. Penalized regression shrinks regression coefficients toward zero, effectively disregarding unimportant predictors.

ML models typically contain one or more main “tuning parameters” that control the complexity of the model. As described in the section on Training/Testing, one can use cross-validation or the bootstrap to select optimal values for these tuning parameters. In general, the more tuning parameters a model has, the more challenging it is to fit the model, since one needs to find optimal values for all tuning parameters which requires searching across a large grid of possible values. Models like CART, random forests, MARS, and penalized regression, often only have fewer tuning parameters that need to be optimized and thus may be considerably easier to implement in practice. In contrast, boosted trees, support vector machines, and neural networks contain more tuning parameters and thus can be more computationally challenging to optimize.

Unlike most ML models, tree-based methods (e.g. CART, random forests, boosted trees) have several additional unique benefits: they are robust to noise/outliers in the predictors [11,79], and they can naturally handle missing values in the predictors using a technique called “surrogate splits” [61]. Although the usual adage of “garbage-in garbage-out” still applies, tree-based methods require minimal data pre-processing of the predictors. For example, most models require standardizing all predictors to be on the same scale, whereas trees can naturally handle a mix of categorical and continuous predictors measured on their original scales, which may be beneficial for EHR data. However, it is worth noting that earlier versions of trees are biased toward favoring categorical variables with more categories and correlated predictors. “Conditional inference trees,” a newer framework for fitting tree-based models, have solved these problems [61,84–86]. Among the models considered in Table 2, neural networks, support vector machines, and boosted trees generally have the longest computation time, followed by random forests. Although rapid advances in computing power and parallel computing means that all of these methods will still be computationally feasible for most applications.

When deciding what ML model to use, it is also important to consider the format/type of outcome that you are trying to predict. Although not listed, all of the models in Table 2 have been extended to handle continuous, binary, or multi-category outcomes. However, ML methods are still rather underdeveloped for handling longitudinal or censored time-to-event (“survival”) outcomes. Some recent work has extended tree-based methods (e.g. CART, random forests) to handle such outcomes [87–89].

Lastly, the models can be compared by how “interpretable” they are. Classical statistical models, CART (“single tree”), MARS, and penalized regression are all easier to interpret. More complex ML models like random forests, boosted trees, support vector machines, and neural networks are much harder to interpret. In general, one can fit a variety of ML models and assess their predictive accuracy using cross-validation. If the cross-validated predictive accuracy of a more interpretable model is comparable to an uninterpretable model, then the more interpretable model should be preferred. The next section discusses tools that can be used to help interpret any ML model.

### Interpreting Black-Box ML Models

ML models are often criticized as being “black-box” models: data are input into a mysterious “black box” which then outputs predictions. However, the user often lacks an explanation or understanding for why the mysterious black box makes a given prediction. Making black-box ML models more interpretable is an ongoing area of research, and we will briefly summarize several important contributions, all of which are implemented in the iml (“interpretable machine learning”) R package [90].

Many ML models produce a type of “variable importance” score [79] for each predictor in the model, allowing the user to rank the predictors from most to least important in their ability to predict the outcome. In addition, one can use “partial dependency plots” [11] (PDPs) to visualize the estimated relationship between the outcome and a specific predictor in the model. For each possible value of the predictor of interest, the PDP will show you the expected value of the outcome, after adjusting for the average effects of all other predictors. For example, one may first rank predictors from most to least important based on their variable importance scores and then use PDPs to visualize the relationship between the outcome and each of the top 5 or 10 most important predictors. Lastly, Friedman’s H-statistic [91] can be used to identify predictors involved in interactions.

Recently, several methods have been developed to help understand why a ML makes a prediction for a particular subject. LIME [92] (“Local Interpretable model explanations”) uses simpler more interpretable models (e.g. linear or logistic regression) to explain how a given subject’s feature values affects their prediction, and the user can control exactly how many features are used to create the “explanation.” The basic idea of LIME is to weigh all subjects by how similar they are to the subject of interest and then fit a simpler interpretable model “locally” by applying these weights. This simpler model is then used to provide an explanation for why the ML model made the prediction for the given subject (or subjects who have features similar to that subject). “Shapley values” [93], originally developed in game theory, are another method for explaining predictions from ML models. Shapley values explain how a subject’s feature values (e.g. gender, age, race, genetics) affect the model’s prediction for that subject compared to the average prediction for all subjects. For example, suppose the model’s average predicted probability of having a particular disease is 0.10. Suppose for a particular subject of interest, “Sam,” the probability of having the disease is 0.60, that is, 0.50 higher than the average prediction. To explain Sam’s prediction, each feature included in the model will get a “Shapley value” that explains how the values of Sam’s features affected the prediction. For example, Sam’s gender increased the prediction by 0.15, Sam’s age increased the prediction by 0.30, and Sam’s race increased the prediction by 0.05. Notice the sum of the Shapley values equals 0.50, which was the difference between Sam’s prediction and the average prediction for all subjects. See Molnar [94] for more information on methods for interpreting ML models.

### Open-Source Software for ML

All of the ML models discussed can be fit within the free open-source software R [95]. The caret R package [96] and tidymodels R package [97] both provide a unified framework for training/tuning and testing over 200 ML models. Table 2 lists a few example R packages for fitting specific ML models. See the “CRAN Task View Machine Learning & Statistical Learning” website [98] for a more comprehensive list of ML R packages. Python also offers free open-source software for ML [99,100].

## Special Considerations of ML in Healthcare Research

### Interpretation

As previously discussed, the “black box” nature of ML methods makes it difficult to understand and interpret the predictions. Although there are some tools that can be used to help interpret ML, further research is still needed for making ML more transparent and explainable. Transparency is especially important when used to make decisions that will impact a patient’s health.

### Fairness/Equity

ML learns from data recorded on past historical examples. However, such historical data can also capture patterns of preexisting healthcare disparities and biases in treatment and diagnosis. ML models trained using such data may further perpetuate these inequities and biases [101]. In addition, if a particular subgroup of patients is under-represented or more likely to have missing data used to train the ML model, then the ML model may produce inaccurate predictions for the subgroup, which can also further exacerbate existing healthcare disparities [102]. Methods and guidelines are being developed to help ensure fairness and equity when using ML models [101–103], but more work is needed. After clinical implementation, ML models should be regularly evaluated for fairness and equity among different minority subgroups. There exist several definitions of fairness and equity within precision medicine that cannot all be simultaneously satisfied, but in general, model evaluation should be conducted for different minority subgroups to ensure that the model performs equally well within each group.

### Need for Translational Prospective Studies Demonstrating Utility of ML

The vast majority of ML in healthcare has been demonstrated using retrospective historical data where outcomes are already known [44–46]. There is a crucial need to demonstrate the utility of ML-based prediction and decision/support tools in real-time prospective studies.

### Legal/Regulatory Issues

If an incorrect medical decision is made based on a complex ML decision support tool, it is unclear what entity should be held liable for that decision [45]. Government regulations for ML-based clinical support tools are still being developed [104].

## Limitations

The authors realize that we have not included mention of several new and important techniques namely in the fields of deep learning, AI, and NLP. We agree that the methods mentioned above are important in the field of predictive modeling and have a place in the analysis of healthcare data. We have chosen to exclude them from this paper as their use is more advanced and requires a more in-depth knowledge of the underlying mathematical and conceptual processes needed to utilize such applications. Deep learning, for instance, involves utilizing multiple layers of neural networks where each network trains a more complicated task. TensorFlow is one example of deep learning and Pytorch is another. Reinforcement learning trains a system to answer multiple questions in sequence. NLP allows data to be extracted from physicians’ notes to be used in analytical applications. All of these are important, recent advances in the field of predictive analytics. Their use, however, is more complex (more training steps, the creation of multiple networks) than the more routine ML process presented above and thus outside the scope of this paper [105,106]. Additionally, the use of AI and NLP in healthcare is still under scrutiny [107,108] and best practices for their use are still being adopted.

## Discussion

ML algorithms have the potential to be powerful and important tools for healthcare research. Here, we have provided researchers with cautionary notes regarding the use of EHR data, insurance databases, and other clinical data with ML algorithms. We have presented the healthcare researcher with a ML pipeline for preparing the data, running the data through various algorithms using separate training/testing datasets, evaluating the created predictive model, tuning any necessary model parameters, and finally validating the predictive model on a separate dataset. In addition, we mention several commonly used ML algorithms frequently referenced in the healthcare literature, and the pros/cons of various supervised methods. Finally, we also mention several considerations for using ML algorithms with healthcare data that, while important, are beyond the scope of this article. Our goal with the concepts and methods described in this article is to educate the healthcare researcher to better understand how to properly conduct a ML project for clinical predictive analytics. We should also note that ML methods do not always perform better than traditional statistical-based methods such as logistic regression [109]. The clinician or translational researcher should familiarize themselves with the differences between traditional statistical-based methods and ML methods and utilize what works best for their specific questions of interest [110].
